# Effects of a Personalized Retrieval-Augmented Generation Chatbot on Information Needs in Patients With Chronic Kidney Disease: Mixed Methods Randomized Controlled Trial

**DOI:** 10.2196/96501

**Published:** 2026-11-13

**Authors:** Hee Jeong Hwang, Junghwan Kim, Dongseok Heo, Jeong Yun You, Jeonghwan Lee, Soie Kwon, Kyung Don Yoo, Jung Han Yoon Park, Bongwon Suh, Jung Pyo Lee, Ki Won Lee

**Affiliations:** 1 Department of Agricultural Biotechnology, College of Agriculture and Life Sciences, Seoul National University, 1, Gwanak-ro, Gwanak-gu, Seoul, Seoul, 08826, Republic of Korea, 82 10-9986-7464; 2 Interdisciplinary Program in Artificial Intelligence, Seoul National University, Seoul, Seoul, Republic of Korea; 3 Department of Internal Medicine, College of Medicine, Seoul National University, Seoul, Seoul, Republic of Korea; 4 Department of Internal Medicine, SNUH SMG-SNU Boramae Medical Center, Seoul, Seoul, Republic of Korea; 5 Department of Internal Medicine, Chung-Ang University Hospital, Seoul, Seoul, Republic of Korea; 6 Department of Internal Medicine, Chung-Ang University, Seoul, Seoul, Republic of Korea; 7 Department of Internal Medicine, Ulsan University Hospital, University of Ulsan College of Medicine, Ulsan, Republic of Korea; 8 Advanced Institutes of Convergence Technology, Seoul National University, Suwon, Gyeonggi-do, Republic of Korea; 9 Center for Food and Bio convergence and Bio-MAX Institute, Seoul National University, Seoul, Seoul, Republic of Korea; 10 Institutes of Green Bio Science & Technology, Seoul National University, Pyeongchang, Gangwon State, Republic of Korea

**Keywords:** renal dialysis, chronic kidney disease, chatbots, retrieval-augmented generation, large language models, self-management, dietary management, information needs, digital health, randomized controlled trial​​​​​​​​​​​​​​​​

## Abstract

**Background:**

Patients receiving dialysis for chronic kidney disease encounter complex self-management challenges and ongoing information needs that traditional health care often fails to meet. Retrieval-augmented generation (RAG) enhances the factual accuracy of large language models (LLMs), but whether personalized RAG is more effective than general RAG remains unclear.

**Objective:**

This study aimed to assess whether personalized constraint retrieval-augmented generation (C-RAG) is more effective than open-domain general retrieval-augmented generation (O-RAG) and standard care in reducing information needs among patients receiving dialysis and to identify predictors of chatbot engagement.

**Methods:**

A sequential 2-phase mixed methods study was conducted. Phase 1 evaluated 4 LLMs (Claude 3.5 Sonnet [Anthropic], Gemini 1.5 Flash [Google], CLOVA X [NAVER], and ChatGPT-4o [OpenAI]) using 60 patient-generated questions, assessed by 15 clinical experts. Phase 2 was a 3-arm parallel pilot randomized controlled trial (RCT; October-December 2025) at 3 tertiary hospitals in South Korea. Based on phase 1 findings, 2 chatbot variants were developed using GPT-4o—C-RAG, which incorporates patient-specific clinical parameters, and O-RAG, which uses the same knowledge base without personalization. Participants were randomized 1:1:1 to C-RAG, O-RAG, or standard care. The primary outcome, change in medication, dietary, and diagnostic information needs (5-point Likert scales) from baseline to 4 weeks, was analyzed using ANCOVA. Semistructured interviews with 9 participants explored underlying mechanisms.

**Results:**

ChatGPT-4o achieved the highest scores in clinical accuracy (mean 3.60, SD 0.95), safety (mean 3.67, SD 1.00), and readability (Flesch-Kincaid Grade Level 9.8, compared with 13.2‐14.5 for other models). In phase 2, 42/45 (93%) enrolled participants completed the study (14 per group). ANCOVA revealed significant group differences in medication (*F*_2,38_=4.574, *P*=.02) and dietary (*F*_2,38_=4.232, *P*=.02) information needs. In Bonferroni-corrected comparisons, C-RAG had lower needs than O-RAG in both domains (observed mean 2.93, SD 1.21 vs mean 3.86, SD 0.95; Cohen *d*=−0.86, 95% CI −1.63 to −0.08; *P*=.02 for both). Against control, C-RAG showed medium-to-large but nonsignificant effects (medication Cohen *d*=−0.64, 95% CI −1.39 to 0.12; dietary Cohen *d*=−0.54, 95% CI −1.30 to 0.21). Secondary outcomes did not differ between groups. Dietary management emerged as the most frequent query topic, accounting for 29.3% (205/691) of total queries. Participants on dialysis for 1‐3 years showed higher engagement (9/10, 90% engagement for 1‐3 y vs 5/18, 27.8% for ≥4 y; Fisher exact *P*=.006). Qualitative analysis revealed 5 themes, including dietary management as the primary need and dialysis duration as a moderator of utility.

**Conclusions:**

In this pilot trial, personalized RAG showed lower information needs than general RAG, with a large effect size (Cohen *d*=−0.86). The medium-to-large effects against standard care (Cohen *d*=−0.54 to −0.64) support a fully powered confirmatory trial (estimated 50‐55 participants per group) to evaluate long-term clinical outcomes.

## Introduction

### Background

Chronic kidney disease (CKD) affects over 850 million people globally [1], with a prevalence exceeding 8% in South Korea [2]. Patients with advanced CKD (stages 3‐5) and those undergoing dialysis face complex self-management challenges, requiring them to simultaneously restrict protein, potassium, phosphorus, sodium, and fluid intake while managing an average of 10‐12 daily medications [3,4]. This complexity results in ongoing information needs, such as “Can I eat this food?” and “When should I take this medication?“—questions that traditional health care delivery often struggles to address [5,6]. For example, a generic recommendation to “increase fluid and potassium-rich fruit intake for general health” can precipitate hyperkalemia or fluid overload in a patient receiving hemodialysis who is anuric, for whom potassium and fluid must instead be restricted. Consistent with this concern, recent work argues that retrieval-grounded conversational systems require evaluation frameworks more rigorous than those applied to generic chatbot outputs, particularly for risk-sensitive clinical content [7].

Current methods of providing information have significant limitations. Educational pamphlets offer generalized content without customization [6], while online search engines may provide unreliable information lacking CKD-specific context [8]. Outpatient consultations, which typically last 5‐10 minutes, cannot provide comprehensive patient education [8,9]. Compounding these structural barriers, clinicians often prioritize acute physiological parameters over patient education during these brief encounters [10-12], and they frequently use inconsistent terminology without verifying patient comprehension [13,14]. This care gap forces patients to navigate complex treatment regimens with inadequate guidance, directly affecting treatment adherence and disease outcomes.

The consequences of insufficient information extend beyond patient frustration. In this study, information needs refer to patients’ self-perceived need for additional disease-related information to support self-management decisions. This construct is distinct from objective knowledge deficits; it captures a subjective state of uncertainty and demand for information, which may persist even when measured knowledge is adequate, and which can be assessed directly by self-report. Patients with CKD report significant unmet information needs regarding medication management, dietary restrictions, and complication prevention [15,16]. The heterogeneous nature of CKD progression and the diverse comorbidity profiles demand highly individualized guidance. Identical questions, such as “Can I eat bananas?” require fundamentally different answers based on dialysis modality, potassium levels, and residual kidney function [17]. Generic information can be not only unhelpful but also potentially harmful when applied inappropriately [18].

### Prior Work

#### Large Language Models in Health Care: Promise and Limitations

Large language models (LLMs) have shown promise in providing 24×7 access to health information through natural language interaction [19,20]. Their ability to process patient queries in conversational language and generate human-like responses positions them as promising tools for addressing the information access gap in health care. However, standard LLMs have significant limitations. They risk hallucination, generating plausible-sounding but factually incorrect information [21], lack transparent reasoning processes, do not provide verifiable citations, and tend to offer generic responses that fail to consider individual patient contexts [22].

Recent evaluations of LLM performance for kidney disease questions showed generally acceptable accuracy in diagnostic and medication domains [23]. However, nutritional guidance—identified as the most common patient concern in social media and dietary-needs studies of patients receiving dialysis—remained problematic across all tested models [16,24]. More fundamentally, existing systems provide population-level information without considering individual patient characteristics that significantly affect appropriate recommendations [17]. This limitation is not just technical; it reflects a conceptual gap—treating personalized medical advice as a generic information retrieval problem.

#### Retrieval-Augmented Generation: Grounding AI in Verified Knowledge

Retrieval-augmented generation (RAG) addresses the limitations of LLMs by grounding responses in verified medical literature and clinical guidelines, thereby enhancing factual accuracy and trustworthiness [25,26]. Rather than relying solely on the parametric knowledge acquired during training, RAG systems first retrieve relevant documents from curated knowledge bases (eg, Kidney Disease: Improving Global Outcomes [KDIGO] guidelines and peer-reviewed nephrology literature) and then generate responses based on this retrieved context. This architecture fundamentally reduces the risk of hallucination while enabling transparent citation of information sources.

Preliminary evidence highlights the effectiveness of RAG in delivering health information. A diabetes education study showed that RAG-enhanced LLMs greatly improved the accuracy and comprehensiveness of responses compared with baseline LLM performance [27]. Similarly, in education of patients with prostate cancer, RAG-based chatbots resulted in higher patient satisfaction and better knowledge retention than traditional static educational materials [28]. However, these studies evaluated generic RAG implementations without addressing the imperative for personalization—the need to tailor information to individual patient parameters.

#### The Critical Gap: Empirical Model Selection and Personalization

Despite the increasing interest in medical AI, model selection in health information systems often occurs without rigorous empirical validation using real patient questions or assessments from domain-specific experts [21]. This represents a critical methodological gap—researchers typically select LLM foundations based on general benchmarks (eg, massive multitask language understanding and reasoning tasks) rather than through systematic evaluation using actual patient-generated queries assessed by clinical domain experts. Such practices risk deploying systems optimized for irrelevant capabilities while underperforming in crucial areas of patient-facing communication, such as readability, safety, and domain-specific accuracy.

Furthermore, while personalized health information systems have shown benefits in managing diabetes and hypertension [29,30], existing approaches typically use rule-based personalization (eg, “IF diabetic THEN restrict sugar”) instead of dynamically integrating comprehensive clinical data. These static personalization strategies lack the flexibility needed to address the complex interactions associated with CKD, where recommendations must consider kidney function, electrolyte levels, dialysis adequacy, medication regimens, and comorbidities simultaneously.

No randomized controlled trials (RCTs) have assessed personalized RAG systems for patients with CKD, leaving the comparative effectiveness of personalized vs generic approaches unestablished. This knowledge gap has significant implications; if personalization offers only marginal benefits, resources should prioritize maximizing accessibility. Conversely, if personalization is found to be transformative, generic systems could do more harm than good by offering misleading guidance with a false sense of authority.

#### Distinguishing Constraint RAG: Technical and Theoretical Contributions

This study introduces the constraint retrieval-augmented generation (C-RAG) system, which offers 2 key innovations that enhance existing approaches. The first innovation is dynamic clinical data integration. Unlike traditional rule-based systems that rely on fixed decision trees, C-RAG dynamically retrieves patients’ relevant clinical parameters—such as current laboratory values, active medications, and dialysis modality. This contextual information is incorporated into both the retrieval query and response generation prompt. As a result, the system can provide responses that take multiple interacting factors into account. For example, it can recommend protein intake based on a patient’s current albumin level, dialysis adequacy (Kt/V, where K is dialyzer urea clearance, t is dialysis session time, and V is the volume of distribution of urea), and the presence of proteinuria.

The second innovation is constraint-based response generation. C-RAG uses explicit constraint checking, ensuring that responses adhere to patient-specific safety boundaries derived from individual clinical data. In contrast to general RAG systems that deliver immediate, generic responses without considering the patient’s context, C-RAG refrains from providing direct answers when critical clinical parameters are absent. For instance, if a patient asks, “Can I eat bananas?” but their serum potassium data are not available, C-RAG will first prompt the patient to provide their recent potassium level before generating dietary guidance. This information-gathering constraint guarantees that responses are tailored to the individual’s clinical context, rather than relying on population-level recommendations that may not be suitable for every patient. More broadly, the constraint-checking and clinical-data integration in C-RAG align with an emerging shift toward auditable, compliance-aware clinical AI, in which generated outputs are separated from a deterministic checking layer and made traceable to patient-specific safety boundaries rather than produced unconditionally [31].

For dietary inquiries, the system calculates allowable ranges for potassium, phosphorus, and protein based on each patient’s parameters, filtering or annotating recommendations as necessary. This constraint mechanism sets C-RAG apart from retrieval-only approaches that simply present general guidelines without considering individual needs. Theoretically, we hypothesize that personalization reduces information needs through two complementary mechanisms: (1) efficiency gains—patients receive directly applicable guidance without the need for iterative questioning to clarify context; and (2) confidence enhancement—an explicit acknowledgment of individual parameters fosters trust and decreases the desire for further verification. This positions the reduction of information needs not only as a measure of satisfaction but also as an indicator of the quality of decisional support—patients need less additional information when initial responses effectively address their specific situations.

### Study Objectives

This mixed methods RCT addressed three primary research questions:

1. Does C-RAG more effectively reduce information needs compared with open-domain general retrieval-augmented generation (O-RAG) and standard care among patients receiving dialysis?
2. What patient characteristics and usage patterns predict engagement with RAG-based chatbots in the population with CKD?
3. How does personalization awareness shape patient information-seeking behaviors, perceived utility, and satisfaction?

We hypothesized that C-RAG would significantly reduce information needs by providing efficient, tailored responses that eliminate the repetitive questioning cycles often seen with generic information. Additionally, we predicted that awareness of personalization would be a key factor influencing the system’s utility, as patients would recognize and appreciate individualized guidance.

The study used a sequential 2-phase design. In phase 1, we systematically evaluated 4 leading LLMs with real patient questions to determine the best foundation model, based on expert assessments of accuracy, safety, and readability. Phase 2 involved a 3-arm RCT that compared C-RAG, O-RAG, and standard care, incorporating qualitative interviews to better understand the mechanisms behind the quantitative outcomes.

## Methods

### Study Design and Overview

We conducted a sequential 2-phase mixed methods study to develop and validate a personalized RAG-based health information system for patients with CKD undergoing dialysis. In phase 1, we used a systematic comparative evaluation framework to identify the optimal LLM foundation by assessing performance in 3 domains—semantic consistency, readability, and clinical validity, using authentic patient-generated questions. Phase 2 involved a 3-arm parallel RCT to assess the clinical effectiveness of C-RAG compared with O-RAG and standard care. This phase also included qualitative interviews to explore the mechanisms behind the quantitative outcomes. This integrated approach facilitated evidence-based progress from technical model selection to clinical implementation while capturing both outcome effectiveness and patient experience.

### Phase 1: Systematic Evaluation and Selection of Foundation Language Model

#### Methodological Framework

Between October and December 2024, we conducted a comprehensive comparative evaluation of 4 leading LLMs to assess their ability to provide accurate, safe, and accessible responses to inquiries from patients with kidney disease in the Korean language context. The complete evaluation workflow is illustrated in Multimedia Appendix 1.

#### Patient Question Collection and LLM Response Generation

We used publicly accessible patient-generated content from KidneyCafe, the largest online kidney disease community in South Korea, which has over 15,000 members. All collected questions were fully anonymized. Since the selected content consisted of general educational inquiries posted publicly without identifiable information, institutional review board (IRB) approval was not required. All identifying elements were removed before analysis. Building on our previously published latent Dirichlet allocation topic modeling research [16], we focused on 3 high-priority clinical domains—examination and diagnostics (laboratory interpretation, symptom evaluation, disease monitoring, and costs); medication and dietary supplements (prescription management, over-the-counter drug safety, and supplement appropriateness); and nutrition and diet (addressing food restrictions, electrolyte management, and dietary guidance for various stages of CKD and dialysis modalities). Within each domain, we selected 20 representative questions (total n=60) based on view count rankings, using high community engagement as a proxy for widespread patient interest. This pragmatic sampling prioritized questions that reflect common patient concerns, although it may underrepresent rare or complex cases with lower engagement.

We evaluated 4 prominent LLMs—Claude 3.5 Sonnet (Anthropic), Gemini 1.5 Flash (Google), CLOVA X (NAVER), and ChatGPT-4o (OpenAI). Each model generated 3 independent responses per question through separate chat sessions conducted on October 23‐October 24, 2024. We ensured strict session isolation to prevent context accumulation. Queries were made using default parameters, without fine-tuning or prompt engineering, to preserve the models’ out-of-the-box performance. Each model was accessed through its publicly available web interface (Claude [Anthropic], ChatGPT [OpenAI], Gemini [Google], and CLOVA X [NAVER]). This approach was chosen to evaluate the baseline capabilities of the models as experienced by typical end users.

#### Semantic Consistency and Readability

We used BERTScore (bidirectional encoder representations from transformers score) to quantify semantic consistency across the 3 independent responses generated by each model. For each model’s 3 responses per question, we conducted pairwise comparisons, resulting in 3 similarity scores. We established a consistency threshold of 0.7 to identify acceptable semantic alignment, based on prior validation studies showing that BERTScore *F*_1_ values above 0.7 indicate substantial semantic similarity [32]. The response with the highest mean BERTScore was selected as the most representative for readability analyses and expert evaluations, focusing on typical outputs while minimizing evaluation burden.

We evaluated text accessibility using 3 complementary readability metrics—the Flesch-Kincaid Grade Level (FKGL), which indicates the years of education required; the Flesch Reading Ease (FRE), which uses a 0‐100 scale where higher scores indicate easier readability; and the Gunning Fog Index (GFI), which estimates the formal education years needed. These metrics were adapted for Korean using the *KoNLPy* library for morphological analysis. To conduct statistical comparisons, we employed the Kruskal-Wallis H test along with Dunn post hoc pairwise comparisons and Bonferroni correction, analyzing both overall model differences and domain-specific performance patterns. Because Flesch-based metrics were originally developed for English, their direct application to Korean is approximate. We used KoNLPy morphological analysis to define word and syllable boundaries appropriate to Korean agglutinative structure, computing syllable counts per morpheme-segmented word and sentence length from analyzer-defined boundaries. These adapted indices were used only for relative comparison across models evaluated on identical Korean inputs, not as absolute grade-level claims; this limitation is noted in the Discussion section.

#### Expert Evaluation of Clinical Accuracy and Safety

We assembled a multidisciplinary panel of 15 board-certified health care professionals, specifically selected to ensure expertise in their respective domains—5 nephrologists, 5 licensed pharmacists, and 5 clinical dietitians. Each evaluator had at least 5 years of specialized clinical experience in managing kidney disease and reported no financial conflicts of interest with AI technology companies.

The expert evaluation used standardized rating protocols to assess the accuracy and safety of responses across 3 clinical domains. For each of the 60 questions, experts reviewed answers from all 4 LLM models as well as community-sourced responses, using two 5-point Likert scales—one for accuracy (1=completely inaccurate, misleading information; 5=completely accurate, comprehensive explanation) and another for safety (1=dangerous, high risk of patient harm; 5=completely safe, appropriate precautions emphasized). Evaluators received standardized instructions outlining the rating criteria, emphasizing the assessment of medical correctness, completeness, appropriateness for the patient audience, and the potential for misinterpretation or harm. To ensure expertise matched to the domain, nephrologists evaluated diagnostic and examination questions, pharmacists evaluated medication and supplement questions, and dietitians evaluated nutrition and diet questions. Each expert independently rated all questions within their domain, resulting in 5 independent ratings per response. Evaluators were blinded to the identity of the models during the assessment, with responses presented in randomized order across evaluation sessions. Community-sourced responses were authentic forum answers and were not length-, context-, or style-matched to the LLM outputs; they were included to represent the real-world peer-information baseline that patients currently encounter. This lack of matching is acknowledged as a limitation when interpreting the community-vs-LLM comparison.

We calculated interrater reliability using Cronbach α to measure expert agreement within each domain. Statistical analysis involved the Kruskal-Wallis H test to evaluate overall differences across the 5 information sources (4 LLMs plus community responses), followed by post hoc pairwise Mann-Whitney U tests with Bonferroni correction when significant omnibus effects were identified. Mean ratings and SDs were computed for each model within each domain and overall, enabling both domain-specific and aggregate performance assessments.

### Phase 2: Clinical Validation of Personalized RAG Through RCT

#### Overview

Phase 2 addressed the central research question—does C-RAG more effectively reduce patient information needs compared with O-RAG and standard care? We conducted a 3-arm parallel-group pilot RCT, incorporating qualitative interviews to establish preliminary efficacy estimates and explore the mechanisms underlying the quantitative outcomes. The overall study design is illustrated in Figure 1, and the characteristics of the 3 study arms are summarized in Table 1.

**Figure 1.**
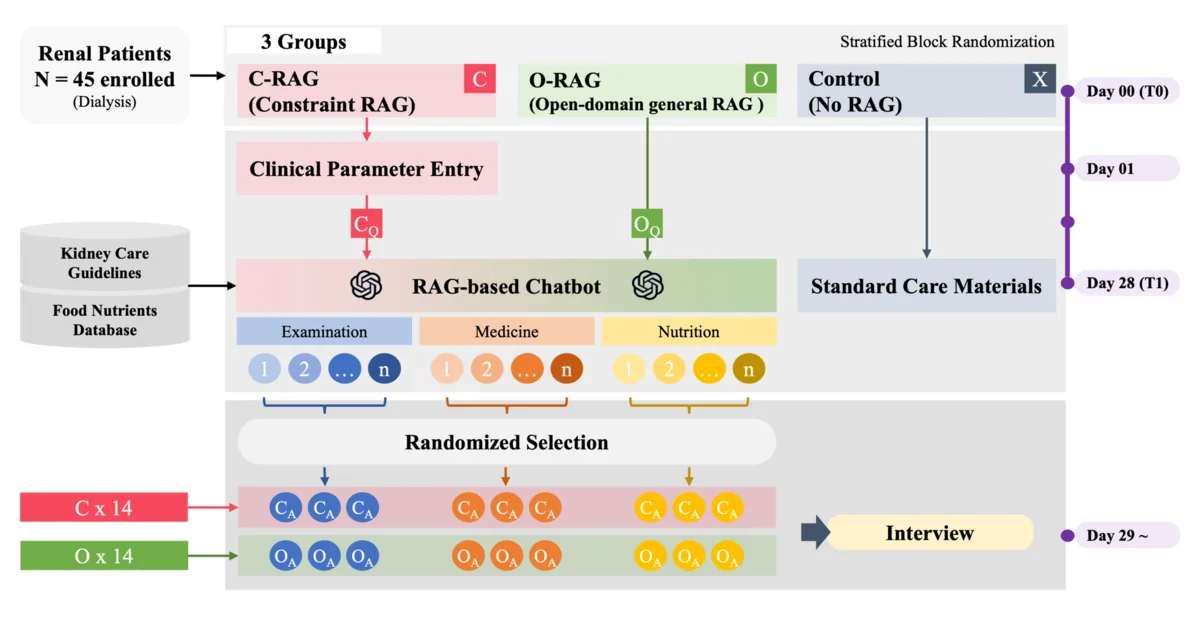
Phase 2 three-arm parallel randomized controlled trial design. Dialysis patients (n=45 enrolled) were randomly assigned in a 1:1:1 ratio to the C-RAG, O-RAG, or standard care control group. Both RAG groups accessed a smartphone-based chatbot grounded in kidney care guidelines and food nutrient databases over a 4-week intervention period. Assessments were conducted at baseline (T0) and 4 weeks (T1). A subset of intervention participants completed semistructured interviews after the intervention period. C-RAG: constraint retrieval-augmented generation; O-RAG: open-domain general retrieval-augmented generation; RAG: retrieval-augmented generation.

**Table 1.** Comparison of study arms: chatbot access, knowledge base, and personalization mechanism. Patient-specific clinical data (laboratory values, comorbidities, and medications) were obtained at enrollment and from patient-uploaded documents via OCR^a^ rather than direct electronic health record access; refer to the Intervention Development and Implementation section for details.

Feature	C-RAG^b^	O-RAG^c^	Control
Chatbot access	Yes	Yes	No
Knowledge base	Shared curated CKD^d^ knowledge base (KDIGO^e^, KDOQI^f^, KDCA^g^, KMA^h^, and KSN^i^ guidelines)	Shared curated CKD knowledge base (KDIGO, KDOQI, KDCA, KMA, and KSN guidelines)	—^j^
Underlying LLM^k^	GPT-4o	GPT-4o	—
Patient-specific data integration	Yes (laboratory values, comorbidities, and medications obtained at enrollment and via OCR of patient-uploaded documents)	No	—
Constraint checking	Yes (retrieved evidence filtered against patient-specific safety rules before response generation)	No	—
Response type	Personalized, patient-bounded recommendations	General educational (population-level) information	Standard care only (no chatbot)

a OCR: optical character recognition.

b C-RAG: constraint retrieval-augmented generation.

c O-RAG: open-domain general retrieval-augmented generation.

d CKD: chronic kidney disease.

e KDIGO: Kidney Disease: Improving Global Outcomes.

f KDOQI: Kidney Disease Outcomes Quality Initiative.

g KDCA: Korea Disease Control and Prevention Agency.

h KMA: Korean Medical Association.

i KSN: Korean Society of Nephrology.

j Not applicable.

k LLM: large language model.

#### Study Setting and Participants

From October to November 2025, we recruited patients receiving dialysis from nephrology outpatient clinics at 3 tertiary medical centers in South Korea—Seoul National University Boramae Medical Center, Ulsan University Hospital, and Chung-Ang University Hospital.

Eligible patients were identified by nephrologists and research nurses during routine outpatient visits at 3 tertiary hospitals (Boramae Hospital, n=26; Ulsan University Hospital, n=9; and Chung-Ang University Hospital, n=10) and approached consecutively. All 45 patients approached were assessed for eligibility, met the inclusion criteria, consented, and were enrolled and randomized; no approached patient declined or was excluded prior to randomization. During follow-up, 3 participants discontinued (C-RAG, n=2, both due to hospitalization for conditions unrelated to the study; O-RAG, n=1, loss of interest; control, n=0), leaving 42 participants (14 per group) for analysis.

Eligibility criteria included age ≥19 years, current maintenance dialysis (hemodialysis or peritoneal dialysis), and having access to a smartphone or computer with basic reading and writing skills suitable for interacting with a text-based system. Basic reading and writing ability was not assessed with a standardized literacy test. Rather, during the consent process, research staff confirmed that each participant could read the study materials and independently complete the self-report questionnaires, which served as the practical criterion for ability to interact with a text-based system. Participants also needed to understand the study procedures and provide written informed consent. Exclusion criteria encompassed cognitive impairments that hindered comprehension of the study content or decision-making ability, limited proficiency in Korean that would restrict questionnaire completion or system use, severe speech or hearing impairments that would prevent participation in interviews, significant comorbidities that would limit participation in the 4-week study, and a history of prior kidney transplantation.

#### Intervention Development and Implementation

Building on GPT-4o, which was selected in phase 1 for its balance of clinical accuracy and readability, we developed 2 RAG-based chatbot variants—a personalized C-RAG and an O-RAG. This 2-variant design was intended to isolate the contribution of patient-specific personalization beyond RAG retrieval alone. This system used a hybrid retrieval pipeline that combined dense embeddings with keyword searches to extract relevant passages from a curated knowledge base. Text embeddings were created using OpenAI embedding models, and documents were indexed in a vector database that supports semantic similarity searches. Retrieved passages were then reranked based on their relevance to the query before synthesizing a response.

The knowledge base included well-established clinical and dietary resources, such as multiple KDIGO clinical practice guidelines (2009‐2024) that cover CKD evaluation, anemia, mineral and bone disorders, diabetes management, blood pressure control, and hepatitis C. It also featured the KDOQI 2020 nutrition guidelines and Korean-language resources from the Korea Disease Control and Prevention Agency, the Korean Medical Association, and the Korean Society of Nephrology. These documents were carefully curated and organized to facilitate the retrieval of evidence pertinent to patient inquiries.

These 2 variants shared the same GPT-4o backbone, retrieval pipeline, and knowledge base, differing only in whether patient-specific constraints were applied. This design allowed attribution of any differential outcome to the personalization mechanism rather than to differences in the underlying model or knowledge source. The C-RAG used patient-specific clinical data—recent laboratory values (eg, serum potassium and phosphorus), comorbidities, and medication information. These data were not drawn from a direct electronic health record interface; instead, they were obtained at enrollment and from patient-uploaded laboratory reports and prescriptions, from which clinical parameters were extracted via optical character recognition (Figure 2B), then used to filter and contextualize responses. Evidence retrieved was subjected to predefined safety rules, and the language model was directed to generate recommendations that adhered to these constraints. To illustrate how the constraint mechanism altered the final answer, consider the query “Can I eat bananas?.” O-RAG returned a general guideline statement that bananas are high in potassium and should be limited in kidney disease. C-RAG, when the patient’s recent serum potassium was unavailable, first requested the value; once a potassium of 5.8 mEq/L was supplied, it advised avoiding bananas and proposed lower-potassium alternatives with portion sizes. A second example involves protein; for “How much meat can I eat?,” O-RAG provided a generic CKD protein range, whereas C-RAG computed a target from the patient’s dialysis modality and recent albumin, yielding a gram-level daily recommendation. Thus, the same retrieved evidence produced population-level guidance in O-RAG but patient-specific, bounded guidance in C-RAG. In contrast, the O-RAG used the same knowledge base and conversational interface but did not incorporate patient-specific constraints, instead offering general educational responses.

**Figure 2.**
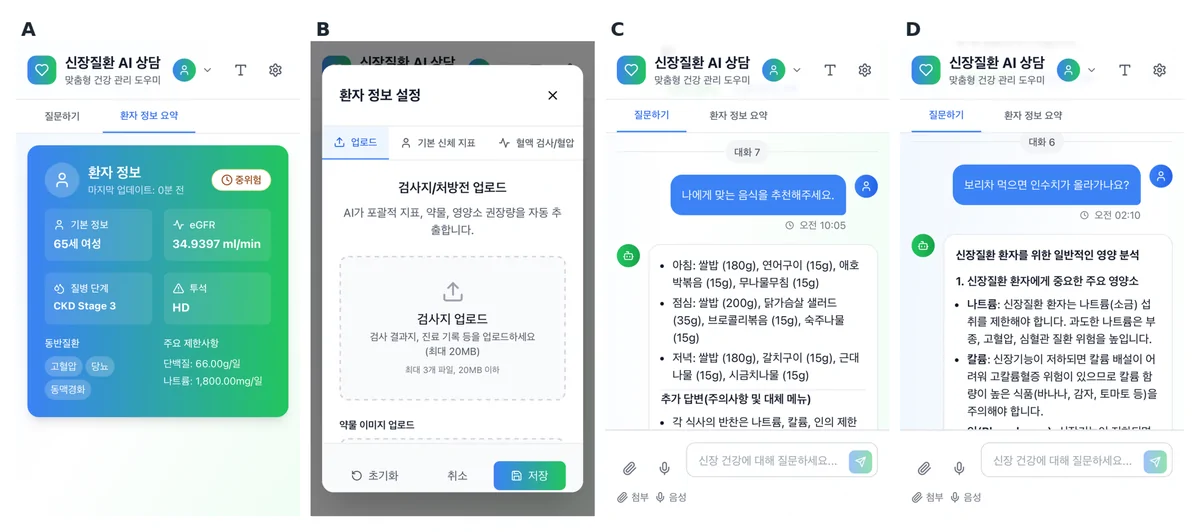
Screenshots of the constraint retrieval-augmented generation (C-RAG) and open-domain general retrieval-augmented generation (O-RAG) chatbot interfaces. (A) The C-RAG patient summary dashboard displaying automatically populated clinical parameters, including estimated glomerular filtration rate, dialysis modality, comorbidities, and personalized dietary restrictions. (B) The patient information settings panel, through which users upload laboratory reports or prescriptions for automated clinical parameter extraction via optical character recognition. (C) A C-RAG response to a dietary query (“please recommend foods suitable for me”), showing a personalized meal plan with gram-level specifications derived from the patient’s individual clinical parameters. (D) An O-RAG response to a dietary query (“does barley tea raise phosphorus levels?"), illustrating a general population-level nutritional response provided without patient-specific contextualization.

The system interface was delivered through a mobile app optimized for older adults, featuring simplified navigation and large text. Participants could submit free-text questions at any time. Responses were generated through a retrieval process followed by language model synthesis, with constraint checks applied before delivery in the C-RAG condition. Representative screenshots of both system interfaces are presented in Figure 2.

Control participants did not have access to the chatbot and continued with standard dialysis care, which included routine nephrologist visits, dietitian consultations, nursing education during dialysis sessions, and patient education materials. This pragmatic control reflects the usual real-world care against which digital health interventions must demonstrate incremental value.

#### Intervention Delivery

Following the baseline assessment and randomization, participants in the C-RAG and O-RAG groups received standardized 30-minute individual training sessions. These sessions covered system access procedures, basic navigation, sample question formulation, and contact information for technical support. The training emphasized the chatbot’s educational purpose and encouraged participants to use it for daily decision-making questions (meal planning, medication concerns, and symptom interpretation). Participants were encouraged to ask at least 3 questions weekly across diagnostic, medication, and dietary domains. This threshold was a pragmatic minimum chosen to ensure at least 1 query per domain per week and thereby meaningful exposure to the system; it was not derived from a formal dose-response rationale. No penalties were applied for not meeting it, and adherence to the threshold was not used as an inclusion or analysis criterion. System access remained available 24×7 throughout the intervention period, with usage patterns tracked through automated logging (timestamp, query text, and response content).

#### Randomization and Allocation Concealment

Randomization sequences were generated by an independent research staff member (not an author) who was not involved in participant recruitment, using computer-generated random numbers with variable block sizes (3 and 6) to minimize predictability in allocation. Allocation concealment was upheld through a centralized web-based registration system, which automatically assigned each participant to a group only after completing enrollment and baseline assessment. This approach ensured that recruitment personnel remained unaware of upcoming assignments. Due to the nature of the intervention, complete blinding was not feasible. Participants were informed of their allocation to either the chatbot group or the control group; however, those in the chatbot groups were not told which specific system they were assigned to (C-RAG vs O-RAG). Research staff responsible for system orientation were unblinded to provide appropriate instruction. Qualitative interview coders remained blinded to group assignments to minimize interpretation bias.

#### Outcome Measures

All outcomes were assessed at baseline (T0) and 4 weeks post randomization (T1), except for the System Usability Scale (SUS), information accuracy, trustworthiness, and clinical utility, which were evaluated at T1 in the intervention groups only. The 4-week time frame provided a balance between meaningful engagement through intervention exposure and the feasibility of participant retention, minimizing burden in this pilot trial.

The primary end point was the reduction in information needs, assessed across 3 clinically relevant domains—medication, dietary, and diagnostic information. Each domain was measured using a single-item 5-point Likert scale (1=no information needed; 5=urgent information needed). The medication information needs following item asked: “How much do you need information about your kidney disease medications (purpose, dosage, side effects, drug interactions)?” The dietary information needs following item asked: “How much do you need information about appropriate foods and eating patterns for your kidney disease?” The diagnostic information needs following item asked: “How much information do you need to understand your laboratory test results and symptoms?” Owing to the absence of a validated instrument for measuring domain-specific information needs in patients with kidney disease [33], single-item Likert scales were used for each domain, consistent with the approach used in prior chatbot RCTs [28].

For secondary outcomes, disease knowledge was assessed using the Kidney Knowledge Survey (KiKS) [15], a validated 28-item instrument that covers CKD-specific knowledge, including kidney function, blood pressure targets, treatment options, symptoms of disease progression, and medications to avoid. Items are scored dichotomously, and total scores are expressed as the number of correct responses (range 0‐28), with higher scores indicating greater disease knowledge. Self-efficacy was measured using the Chronic Kidney Disease Self-Efficacy scale [34], a 25-item instrument rated on a 5-point Likert scale (1=not at all confident; 5=very confident). Total scores range from 25 to 125, with higher scores indicating stronger self-efficacy. Treatment adherence was assessed across 3 domains—medication adherence, dietary adherence, and fluid restriction adherence. Medication adherence was evaluated using the 8-item Morisky Medication Adherence Scale (MMAS-8) [35], which comprises 7 yes or no items and one 5-point item (total score 0‐8; higher scores indicate better adherence). Dietary adherence and fluid restriction adherence were each assessed using a single 5-point Likert item (1=not at all adherent; 5=very adherent).

In the intervention groups only, system usability was evaluated at T1 using the SUS, a validated 10-item instrument that yields scores from 0 to 100. Scores of 68 or above indicate acceptable usability [36]. Additionally, three 5-point Likert items assessed: (1) satisfaction with the preparation and quality of clinical consultations, (2) satisfaction with the quality of health information provided by the chatbot, and (3) trust in the health information delivered by the chatbot. Usage patterns were captured through automated system logs that recorded query frequency, temporal distribution (day of the week, hour of the day), and topic classification (diagnostic, medication, dietary, lifestyle, and general).

#### Qualitative Interview Substudy

To enhance the understanding of quantitative outcomes and the mechanisms behind observed effects, we conducted semistructured interviews with a purposive sample of 9 participants (5 from C-RAG and 4 from O-RAG) chosen to maximize variation in engagement levels. This pragmatic sample size was influenced by resource constraints from the pilot trial, while still ensuring representation across both intervention arms and usage patterns.

The interviews were held one-on-one by 2 research team members (HJH and JYY) using a semistructured guide. This guide focused on overall system experience and perceived usefulness, specific examples of information-seeking behaviors, the impact of responses on decision-making, comparisons with alternative information sources (clinicians, internet, and peer communities), and suggestions for improvement. The full semistructured interview guide is provided in Multimedia Appendix 2.

With participant consent, the interviews were audio-recorded and transcribed verbatim by team members. Thematic analysis was conducted following Braun and Clarke’s 6-phase framework—familiarization, initial coding, theme development, theme review, theme refinement, and report production [37]. Given the small purposive sample tied to the pilot design, we did not conduct a formal data-saturation assessment; coding continued until the 2 coders (HJH and JYY) reached consensus, and the limited sample is acknowledged as a constraint on thematic completeness. The 2 researchers (HJH and JYY) independently coded the transcripts, resolved discrepancies through discussion, and identified themes through an iterative comparison process.

### Statistical Analysis

#### Sample Size and Power Considerations

This study was designed as a pilot RCT, with the primary objective of estimating effect size to inform a future fully powered trial rather than conducting definitive hypothesis testing [38]. Accordingly, between-group comparisons—except the better-powered C-RAG vs O-RAG contrast—are interpreted as effect-size estimates to inform a future confirmatory trial, rather than as definitive tests of efficacy. A target enrollment of 20 participants per group (totaling 60) was established based on G*Power 3.1 (Heinrich Heine University Düsseldorf) calculations for 1-way ANCOVA with 3 groups (80% power, α=.05, 1 covariate, Cohen *f*=0.35, pre-post correlation of 0.65), accounting for a 15% anticipated attrition rate. Given the pilot design, the findings should be interpreted as preliminary estimates rather than confirmatory evidence.

#### Analytic Approach

Group differences at 4 weeks were assessed using 1-way ANCOVA, incorporating the corresponding baseline score as a covariate. When the omnibus test was significant, pairwise comparisons were conducted using contrast *t* tests within the same ANCOVA model to ensure consistent baseline adjustment, applying Bonferroni correction for 3 comparisons. Effect sizes are reported as Cohen *d*, interpreted as small (|Cohen *d*|≥0.2), medium (|Cohen *d*|≥0.5), large (|Cohen *d*|≥0.8), and very large (|Cohen *d*|≥1.2). Missing data were addressed through complete-case analysis. Usage patterns were summarized descriptively. All analyses were performed in Python (version 3.11.5; Python Software Foundation) with a 2-tailed α=.05.

### Ethical Considerations

This study was conducted in accordance with the Declaration of Helsinki. The protocol received IRB approval at all 3 clinical sites (IRB 10-2025-62, IRB 2510-003-660, and UUH IRB 2025-09-025) and at Seoul National University for data analysis (IRB 2508/002‐001). All participants provided written informed consent, which included explicit notification that their medical care would not be affected if they chose to withdraw at any time. The capacity to provide informed consent was verified prior to enrollment. The phase 1 online community data were exempt from IRB approval, as no individually identifiable information was used. The phase 2 study was registered in the Clinical Research Information Service (registration KCT0011656).

## Results

### Phase 1: LLM Selection Through Systematic Evaluation (ChatGPT-4o)

#### Semantic Consistency and Readability

All 4 evaluated LLMs demonstrated acceptable semantic consistency, with BERTScore values exceeding the 0.7 threshold across the 3 independent responses generated per question. ChatGPT-4o achieved superior readability in all 3 metrics (FKGL 9.8, FRE 62.4, and GFI 11.2), indicating a high-school reading level, compared with Claude, Gemini, and CLOVA X (FKGL 13.2‐14.5, college-level). The Kruskal-Wallis H test confirmed significant differences in readability across the models (*P*<.001; Multimedia Appendix 3 ).

#### Expert Evaluation of Clinical Accuracy and Safety

Interrater reliability was strong across all evaluations (Cronbach α 0.802‐0.984). ChatGPT-4o achieved the highest clinical accuracy (mean 3.60, SD 0.95) and safety ratings (mean 3.67, SD 1.00), followed by Gemini, which had an accuracy mean of 3.54 (SD 1.07) and a safety mean of 3.62 (SD 1.08). There was no significant difference between ChatGPT-4o and Gemini (Mann-Whitney *U* test). Claude demonstrated intermediate performance with an accuracy mean of 3.32 (SD 1.12) and a safety mean of 3.45 (SD 1.15), while CLOVA X showed lower accuracy (mean 3.08, SD 1.18). All 4 LLMs substantially outperformed community-sourced responses from KidneyCafe patient forums, which had an accuracy mean of 2.49 (SD 1.21) and a safety mean of 2.44 (SD 1.28; Kruskal-Wallis H test, *P*<.001). Domain-specific analysis revealed that all models performed comparably in the inspection or diagnostics and medications or supplements domains, while the nutrition and diet domain yielded lower scores across all models (Figure 3).

**Figure 3.**
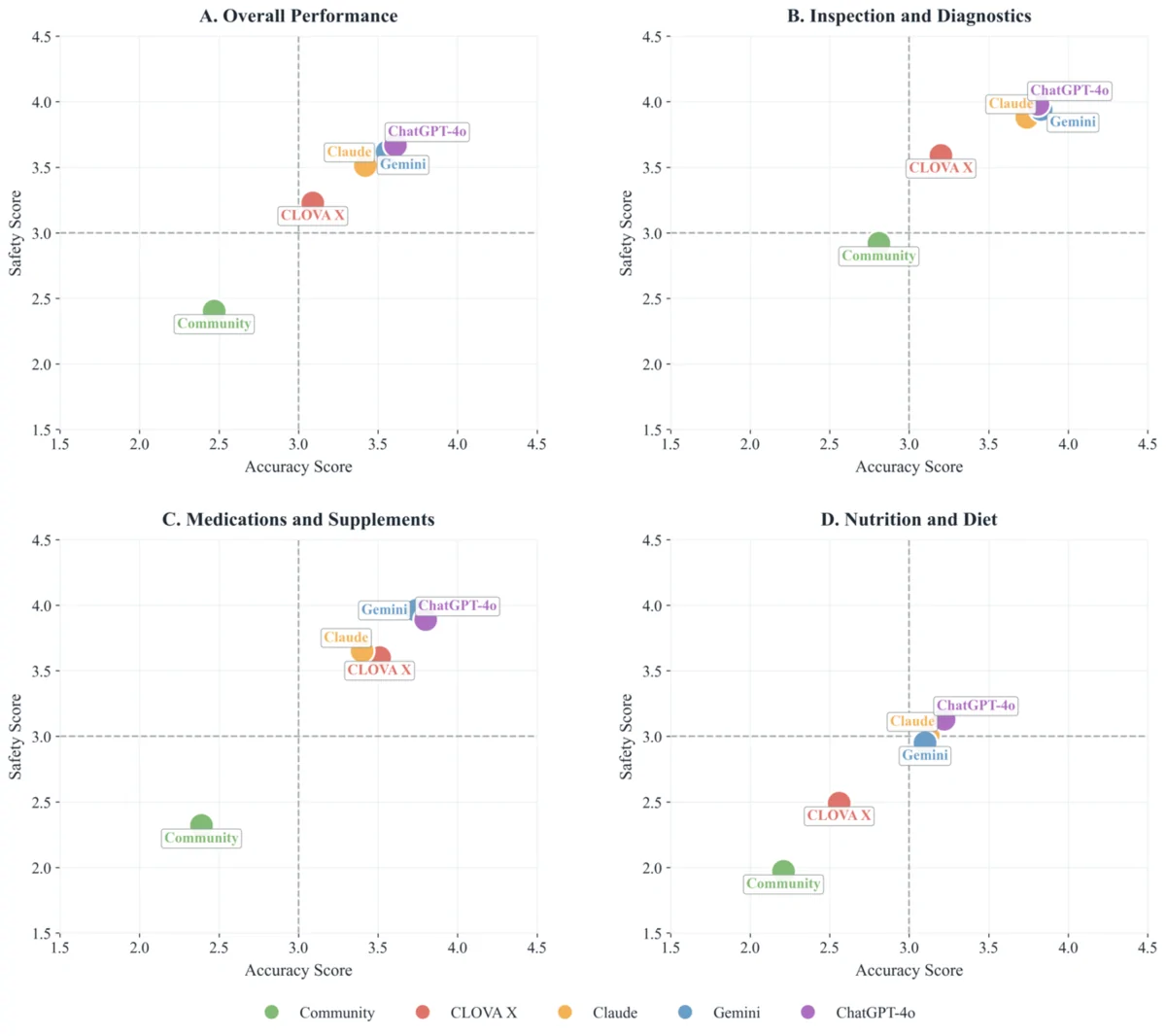
Clinical accuracy and safety scores across large language models (LLMs) by domain. Each panel displays the mean accuracy (x-axis) and safety (y-axis) scores for 4 LLMs and community-sourced responses. Dashed lines indicate score 3.0 (acceptable threshold). ChatGPT-4o and Gemini consistently occupied the high-accuracy, high-safety quadrant across all domains. The nutrition and diet domain exhibited the lowest overall performance among all models, including ChatGPT-4o. Community-sourced responses consistently fell below the acceptable threshold in both accuracy and safety dimensions.

#### Model Selection

ChatGPT-4o’s combination of top-tier clinical validity (accuracy mean 3.60, SD 0.95; safety mean 3.67, SD 1.00), superior patient accessibility (FKGL 9.8 vs 13‐14 for competitors), and consistent cross-domain performance established it as the optimal foundation model. While Gemini demonstrated comparable clinical scores, ChatGPT-4o’s 4-grade-level readability advantage makes it more appropriate for diverse patient populations with varying educational backgrounds. ChatGPT-4o was therefore selected for phase 2 RAG system development.

### Phase 2: Clinical Validation of Personalized RAG Through RCT

#### Participant Flow and Baseline Characteristics

Between October and November 2025, 45 dialysis patients were evaluated for eligibility across 3 hospital sites. After randomization, 3 participants withdrew from the intervention period—2 from the C-RAG due to hospitalization for unrelated conditions and one from the O-RAG due to loss of interest—resulting in 42 completers (14 per group; 93% retention rate).

The incidence of withdrawal was consistently low across all study groups (C-RAG 2/16, O-RAG 1/15, and control 0/14) and was unrelated to the intervention, supporting a complete-case analysis. Although enrollment fell short of the planned 60 participants due to recruitment constraints at various sites during the study period, the achieved sample aligns with precedents from pilot RCTs in CKD digital health interventions [27,28] and is sufficient for effect size estimation. All 42 completers provided usable data for analysis (Figure 4).

**Figure 4.**
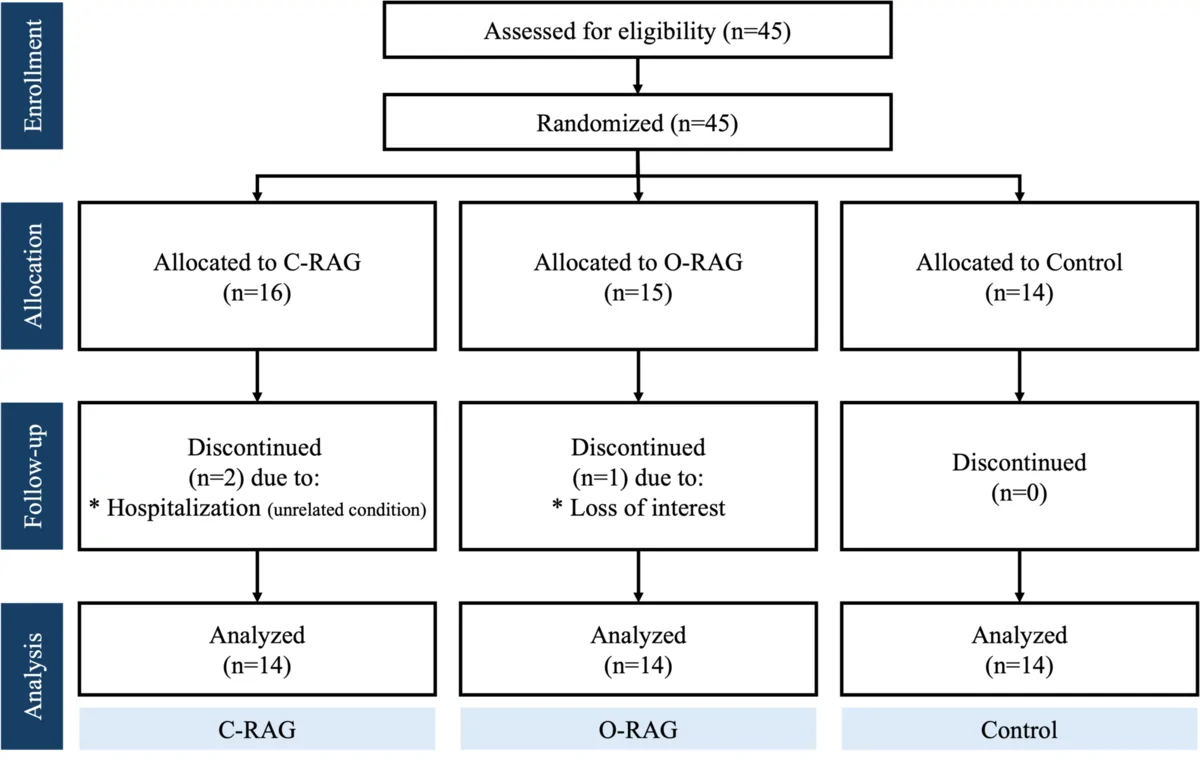
CONSORT (Consolidated Standards of Reporting Trials) flow diagram of participant enrollment, randomization, and analysis. C-RAG: constraint retrieval-augmented generation; O-RAG: open-domain general retrieval-augmented generation.

Baseline characteristics revealed no statistically significant differences among the groups (Table 2). Age distribution was comparable (*P*=.70), with the majority of participants in their 40s-50s across all groups. Sex distribution showed male predominance (12/14, 85.7% C-RAG; 12/14, 85.7% O-RAG; and 11/14, 78.6% control; *P*=.84). Dialysis modality was predominantly hemodialysis across all groups (13/14, 92.9% C-RAG; 14/14, 100% O-RAG; and 14/14, 100% control), with 1 C-RAG participant on peritoneal dialysis (*P*≥.99). Dialysis duration distribution was comparable across groups (*P*=.50), with the majority of participants having ≥5 years of dialysis experience. Comorbidity prevalence was comparable across groups—diabetes mellitus (*P*=.08), hypertension (*P*=.10), and cardiovascular disease (*P*=.86). Baseline kidney knowledge, self-efficacy, information needs, and adherence measures showed no significant differences across groups (*P*=.07‐.82).

**Table 2.** Baseline characteristics of participants by group.

Characteristic	C-RAG^a^ (n=14)	O-RAG^b^ (n=14)	Control (n=14)	*P* value^c^
Age (y), n (%)				.70
40s	4 (28.6)	2 (14.3)	4 (28.6)	
50s	4 (28.6)	8 (57.1)	4 (28.6)	
60s	2 (14.3)	0 (0)	2 (14.3)	
70s	2 (14.3)	3 (21.4)	3 (21.4)	
80+	2 (14.3)	1 (7.1)	1 (7.1)	
Sex, n (%)				.84
Male	12 (85.7)	12 (85.7)	11 (78.6)	
Female	2 (14.3)	2 (14.3)	3 (21.4)	
Dialysis modality, n (%)				≥.99
Hemodialysis	13 (92.9)	14 (100)	14 (100)	
Peritoneal dialysis	1 (7.1)	0 (0)	0 (0)	
Dialysis duration, n (%)				.50
<6 months	2 (14.3)	0 (0)	0 (0)	
6 months-1 year	1 (7.1)	2 (14.3)	1 (7.1)	
1-3 years	2 (14.3)	3 (21.4)	1 (7.1)	
3-5 years	4 (28.6)	2 (14.3)	3 (21.4)	
≥5 years	5 (35.7)	7 (50)	9 (64.3)	
Comorbidities, n (%)				
Diabetes mellitus	10 (71.4)	4 (28.6)	7 (50)	.08
Hypertension	10 (71.4)	13 (92.9)	8 (57.1)	.10
Cardiovascular disease	2 (14.3)	3 (21.4)	3 (21.4)	.86
Baseline measures, mean (SD**)**				
Kidney knowledge (KiKS^d^)	18.6 (3.4)	19.6 (3.1)	19.0 (3.7)	.82
Self-efficacy (CKD-SE^e^)	81.4 (12.9)	86.7 (14.1)	86.8 (16.2)	.58
Diagnosis info needs	3.2 (1.1)	3.2 (1.0)	3.4 (1.2)	.81
Medication info needs	3.8 (0.8)	2.9 (1.1)	3.6 (1.2)	.07
Diet info needs	3.6 (1.2)	3.3 (1.1)	3.9 (1.1)	.38
Medication adherence (MMAS-8^f^)	6.2 (1.4)	5.7 (2.0)	6.0 (2.0)	.69
Diet adherence	2.6 (0.7)	3.4 (1.0)	3.1 (0.7)	.08
Fluid adherence	2.9 (0.8)	3.4 (1.0)	2.9 (0.5)	.07

a C-RAG: constraint retrieval-augmented generation.

b O-RAG: open-domain general retrieval-augmented generation.

c *P* values were calculated using the Kruskal-Wallis test for continuous variables and the chi-square test or Fisher exact test for categorical variables.

d KiKS: Kidney Knowledge Survey.

e CKD-SE: Chronic Kidney Disease Self-Efficacy Scale.

f MMAS-8: Morisky Medication Adherence Scale-8.

#### Primary Outcomes: Information Needs

A 1-way ANCOVA adjusting for baseline scores revealed significant group differences in medication information needs (*F*_2,38_=4.574, *P*=.02) and dietary information needs (*F*_2,38_=4.232, *P*=.02) at 4 weeks. No significant group difference was found for diagnostic information needs (*F*_2,38_=0.835, *P*=.44; Table 3).

**Table 3.** Primary outcomes: information needs at 4 weeks. Values are observed means at 4 weeks (SD) on a 1‐5 Likert scale; lower scores indicate lower information needs.

Domain and comparison	C-RAG^a^ , mean (SD)	O-RAG^b^, mean (SD)	Control, mean (SD)	Effect size, Cohen *d^c^* (95% CI)	*P* value
Medication	2.93 (1.21)	3.86 (0.95)	3.71 (1.27)		
ANCOVA^d^				—^e^	.02
C-RAG vs O-RAG				−0.86 (−1.63 to −0.08)	.02
C-RAG vs Control				−0.64 (−1.39 to 0.12)	.13
O-RAG vs Control				0.13 (−0.61 to 0.87)	≥.99
Dietary	2.93 (1.21)	3.86 (0.95)	3.57 (1.16)		
ANCOVA^d^				—	.02
C-RAG vs O-RAG				−0.86 (−1.63 to −0.08)	.02
C-RAG vs Control				−0.54 (−1.30 to 0.21)	.55
O-RAG vs Control				0.27 (−0.47 to 1.01)	.40
Diagnostic	3.43 (1.22)	3.93 (0.83)	3.71 (1.07)		
ANCOVA^d^				—	.44
C-RAG vs O-RAG				−0.48 (−1.23 to 0.27)	.61
C-RAG vs Control				−0.25 (−0.99 to 0.50)	≥.99
O-RAG vs Control				0.22 (−0.52 to 0.97)	≥.99

a C-RAG: constraint retrieval-augmented generation.

b O-RAG: open-domain general retrieval-augmented generation.

c Cohen *d* calculated from raw observed group values.

d ANCOVA omnibus test (*F*_2,38_) adjusted for corresponding baseline score. Pairwise comparisons via contrast *t* tests within ANCOVA model with Bonferroni correction for 3 comparisons. Medication and dietary information-needs scores were highly correlated within participants (*r*=0.49‐0.85 across groups, including at baseline), reflecting a general tendency for patients to rate these closely related domains similarly; the resulting identical group-level means, SDs, and effect sizes are a consequence of this correlation pattern rather than a data-entry artifact.

e Not applicable.

Pairwise comparisons using Bonferroni-corrected contrast *t* tests within the ANCOVA model indicated that C-RAG participants had significantly lower information needs than O-RAG participants at 4 weeks for both the medication (Cohen *d*=−0.86, 95% CI −1.63 to −0.08; *P*=.02) and dietary domains (Cohen *d*=−0.86, 95% CI −1.63 to −0.08; *P*=.02), representing large effect sizes. No significant differences were found between C-RAG and the control group, or between O-RAG and the control group, for any domain (medication Cohen *d*=−0.64, 95% CI −1.39 to 0.12; dietary Cohen *d*=−0.54, 95% CI −1.30 to 0.21).

Examination of change scores revealed divergent trajectories between the two RAG groups (Figure 5). C-RAG participants demonstrated reductions in information needs from baseline in both the medication (Δ=−0.87) and dietary (Δ=−0.67) domains, indicating improved fulfillment of information needs. In contrast, O-RAG participants experienced increases in information needs in both domains (Δ=+0.96 and Δ=+0.56, respectively), although these increases did not differ significantly from the control group. This pattern suggests a potential trend toward heightened awareness of unmet information needs, although the lack of statistical significance limits definitive interpretation. Control participants exhibited minimal change across all domains.

**Figure 5.**
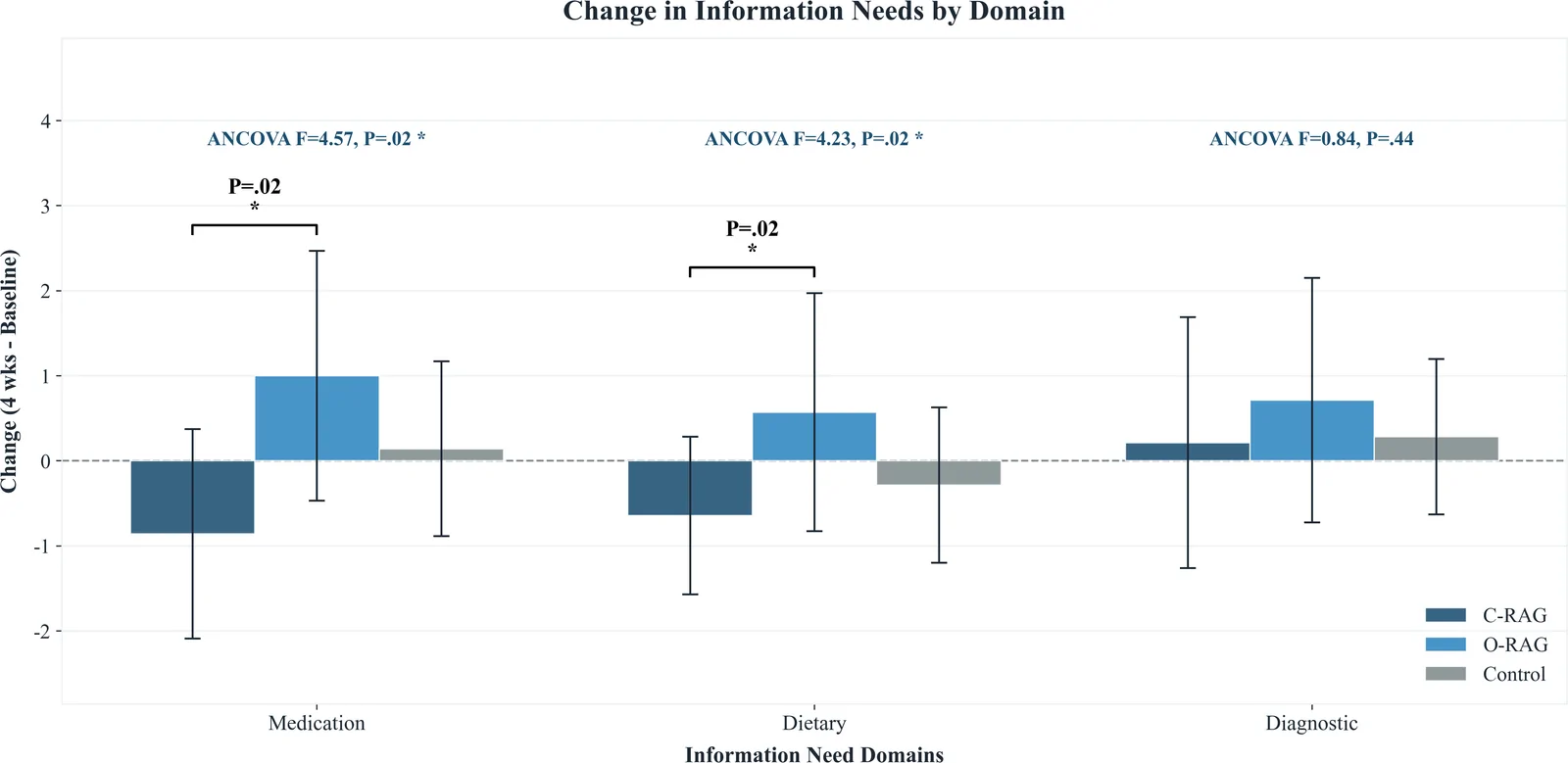
Information needs change scores by domain and group. Constraint retrieval-augmented generation (C-RAG) demonstrated large effect sizes for information needs reduction vs open-domain general retrieval-augmented generation (O-RAG) in medication (Cohen *d*=−0.86, *P*=.02) and dietary domains (Cohen *d*=−0.86, *P*=.02). Error bars represent standard deviation. *P<.05, trend (*P*<.10).

#### Usability and Satisfaction

In the intervention groups, the mean SUS score was 62.50 (SD 15.60) for C-RAG and 66.96 (SD 14.91) for O-RAG, both marginally below the 68-acceptability threshold, with no significant between-group difference (*P*=.55). Satisfaction with consultation preparation or quality (C-RAG mean 3.50, SD 0.65; O-RAG mean 3.79, SD 1.12; *P*=.24), chatbot information quality (mean 3.64, SD 0.74 vs mean 3.79, SD 0.70; *P*=.74), and trust in chatbot information (mean 3.57, SD 0.65 vs mean 3.86, SD 0.66; *P*=.35) likewise did not differ between groups.

#### Secondary Outcomes

Secondary outcomes including kidney disease knowledge (KiKS), self-efficacy (Chronic Kidney Disease Self-Efficacy scale), and treatment adherence showed no significant group differences over the 4-week intervention period (Multimedia Appendix 4), consistent with the short duration of this pilot study.

#### Chatbot Usage Patterns

System logs provided comprehensive usage data from 28 intervention participants (14 C-RAG and 14 O-RAG) throughout the 4-week study period, offering objective behavioral evidence of how patients integrated the chatbot into their daily disease management routines.

Participants generated 691 questions collectively, yielding a mean of 24.68 (SD 24.29) questions per participant (median 13.0, IQR 4.0‐40.0). Usage frequency demonstrated marked heterogeneity, with participants clustering into 2 distinct engagement profiles. Because no validated cutoff for chatbot engagement exists, participants were dichotomized at the sample median (13.0 questions) into higher- and lower-engagement groups for exploratory within-sample comparison only. Higher-engagement users (n=14) averaged 44.8 (SD 18.5) questions, whereas lower-engagement users (n=14) averaged 4.6 questions. C-RAG and O-RAG groups showed comparable usage levels (mean 20.00 (SD 20.15) vs mean 29.36 (SD 27.79) questions; *P*=.40), indicating that personalization affected information quality rather than engagement quantity.

To identify patient populations most likely to benefit from chatbot interventions, we conducted an exploratory analysis comparing higher- and lower-engagement users (Table 4). Dialysis duration emerged as the only significant predictor (*P*=.006)—9 of 10 (90%) patients with shorter dialysis duration (1‐3 y) showed high engagement vs 5 of 18 (27.8%) with longer duration (≥4 y). Sex showed no significant association with engagement (*P*=.11). Age, dialysis modality, diabetes status, digital device proficiency, and prior chatbot experience were not associated with engagement (Table 4), suggesting broad accessibility regardless of technical background.

**Table 4.** Predictors of high chatbot engagement.

Characteristic	High engagement^a^ (n=14)	Low engagement (n=14)	*P* value^b^
Sex, n (%)			.11
Female	4 (100)	0 (0)	
Male	10 (41.7)	14 (58.3)	
Dialysis duration (y), n (%)			.006
1-3	9 (90)	1 (10)	
≥4	5 (27.8)	13 (72.2)	
Dialysis modality, n (%)			≥.99
HD^c^	14 (51.9)	13 (48.1)	
PD^d^	0 (0)	1 (100)	
Diabetes mellitus, n (%)			.71
Yes	6 (42.9)	8 (57.1)	
No	8 (57.1)	6 (42.9)	
Digital device proficiency, mean (SD)	3.4 (1.0)	3.3 (0.8)	.64
Prior chatbot experience, n (%)			.45
Yes	8 (61.5)	5 (38.5)	
No	6 (40)	9 (60)	

a High engagement defined as above the median number of questions submitted during the 4-week intervention period (median 13.0, range 0‐89). Analysis restricted to C-RAG and O-RAG groups (n=28).

b *P* values were calculated using the Fisher exact test for categorical variables and the Mann-Whitney *U* test for continuous variables.

c HD: hemodialysis.

d PD: peritoneal dialysis.

#### Information-Seeking Priorities and Temporal Integration

Topic distribution analysis (Multimedia Appendix 5A) showed that dietary queries comprised the largest single category (205/691, 29.3%), followed by diagnostic (156/691, 22.6%) and medication (122/691, 17.7%) queries; keyword-level frequencies are provided in Multimedia Appendix 5B.

Temporal usage revealed distinct patterns; engagement peaked in week 1 (276 questions) and tapered toward week 4 (107 questions), and was higher on weekdays than weekends, peaking Tuesday through Thursday (Multimedia Appendix 5C). Hourly analysis identified morning (9 AM) and evening (9 PM) peaks that were dietary-dominant, whereas midday peaks (2 PM‐3 PM) showed a more varied mix of diagnostic and lifestyle queries (Multimedia Appendix 5D).

### Qualitative Findings: Personalization Awareness Fundamentally Transforms Patient Experience

#### Overview

A total of 9 patients receiving dialysis completed semistructured interviews at the study end point—5 from the C-RAG group and 4 from the O-RAG group. Table 5 presents the participant characteristics, including dialysis duration and levels of system engagement. Moreover, 5 major themes emerged from the analysis, providing mechanistic insight into quantitative outcome patterns.

**Table 5.** Characteristics of qualitative interview participants. System usage categories based on total number of sessions during the 4-week intervention period: minimal (1-5), low (6-15), moderate (16-50), high (>50)

ID	Group	Age group (y)	Sex	Dialysis duration	Diagnosis duration	System usage and total session count
O1	O-RAG^a^	50‐59	Female	≥5 years	≥10 years	High (54)
C3	C-RAG^b^	30‐49	Male	≥5 years	≥10 years	Low (7)
O4	O-RAG	65‐74	Male	6 months-1 year	≥10 years	High (89)
C5	C-RAG	65‐74	Male	≥5 years	5‐10 years	Moderate (45)
C16	C-RAG	50‐59	Female	6 months-1 year	1‐3 years	Moderate (33)
O19	O-RAG	30‐49	Male	≥5 years	5‐10 years	Low (12)
O28	O-RAG	50‐59	Male	≥5 years	5‐10 years	Minimal (1)
C29	C-RAG	60‐64	Male	3‐5 years	5‐10 years	Low (4)
O30	O-RAG	50‐59	Male	3‐5 years	5‐10 years	Low (4)

a O-RAG: open-domain general retrieval-augmented generation.

b C-RAG: constraint retrieval-augmented generation.

Although the 5 themes emphasize perceived value, participants also voiced limitations and improvement requests. These included the burden of repeatedly providing clinical context in O-RAG, diminished usefulness for patients receiving long-term dialysis who found responses redundant, and a preference for automatic contextualization. A small number of participants also reported occasional technical issues, such as response delays or repetition of previously provided information, although these were not identified as a dominant theme. We report these alongside the positive accounts to avoid an overly favorable portrayal.

#### Theme 1: Intrinsic Value of Disease-Specific Chatbot Access

Participants in both groups expressed significant appreciation for immediate access to kidney disease information through the chatbot system, regardless of personalization features. This baseline value was reflected in temporal accessibility, disease domain specificity, and a reduced burden on clinical staff.

*The answers were helpful, so I continued using it. I could rely on it every time I ate. Its immediate responses—whether I was starting to eat or applying medication—made it very convenient to use.*

[O4]

*It provided more detailed answers than what doctors typically offer, which was both comfortable and beneficial. The ability to ask any questions was very helpful and convenient.*

[O1]

Both groups expressed a strong desire to continue using the disease-specific AI assistants after the study, indicating that these tools address fundamental structural limitations in health care delivery.

#### Theme 2: Varied Perception of Personalization Independent of System Design

Participant perceptions of whether responses were personalized showed substantial variability within both groups and did not consistently align with actual system assignments. Some O-RAG users perceived responses as personalized, despite the generic system design, suggesting that motivated users achieved functional personalization by initiating context in their queries.

*I recognized it as personalized. When I asked whether I could eat spinach and how much, it responded with the recommended grams to consume and detailed the components, aligning with my information.*

[O1]

However, O-RAG users reported that achieving this specificity required providing clinical context with each query, and they noted that automatic contextualization would have alleviated this burden. This automatic contextualization, observed qualitatively, parallels the quantitative finding that C-RAG reduced information needs while O-RAG did not, suggesting that the interaction burden of manual contextualization is one mechanism underlying the divergent change scores.

*Even without a personalized system, explaining that I was on dialysis before asking allowed me to receive tailored answers. However, it would have been more convenient if the chatbot had already known my situation.*

[O1]

One plausible interpretation is that repeated exposure to generic answers, which did not resolve patients’ specific concerns, generated dissatisfaction and prompted further questioning—heightening awareness of unmet needs rather than fulfilling them. The high-frequency O-RAG user who attributed elevated usage to iterative follow-up is consistent with this account. Because the O-RAG vs control difference did not reach significance, this remains a hypothesis to be tested in larger samples. One high-frequency O-RAG user (89 questions) explicitly attributed his elevated usage volume to the need for iterative query refinement:

*Many responses were general, so I needed to ask more specific follow-up questions to obtain answers that matched my situation, which increased the total number of questions.*

[O4]

C-RAG participants, in contrast, recognized personalization features when the system referenced their pre-entered clinical data without needing additional prompts:

*At the beginning of each response, the chatbot displayed my health status first, which made me feel that my condition was accurately reflected in the answers.*

[C16]

This automatic contextualization reduced the interaction burden needed to obtain clinically relevant responses.

#### Theme 3: Dietary Management as Predominant Information Need

Of the nine interview participants, 8 identified dietary management as their most important and frequently addressed information need, regardless of group assignment. This universal emphasis highlights the temporal mismatch between the frequency of dietary decisions and the availability of clinical dietary guidance. This aligns with quantitative results showing large effect size differences between C-RAG and O-RAG in the dietary information need domain (Cohen *d*=−0.86, *P*=.02). This convergence between participants’ lived experience and the large observed quantitative effect (Cohen *d*=−0.86) reinforces dietary guidance as both the area of greatest unmet need and the domain of greatest intervention benefit.

*Dietary management was the hardest to manage. Even now, it’s not resolved, and the diet is really difficult. It helped with dietary management, and it was good that it told me exactly what not to eat.*

[C3]

*Dietary management was the most challenging aspect. I had many foods I wanted to enjoy, but I was always worried about portion sizes and the phosphorus and potassium content of those foods. Finding a way to address these concerns proved to be very helpful.*

[O1]

#### Theme 4: Dialysis Experience Duration as Dominant Moderator of Utility

The most notable pattern observed in both intervention arms was the inverse relationship between dialysis duration and perceived chatbot utility. Patients with extensive dialysis experience (>3 y) reported significantly limited benefits, regardless of group assignment, while those with shorter experience found considerable value in both systems.

*Having been on dialysis for 5 years, I already knew a lot, so my usage wasn’t high. Only information I was familiar with was presented, which was disappointing. However, it seems very necessary for those who haven’t been on dialysis for long.*

[C29, C3]

This pattern suggests that chatbot interventions focus on a specific phase of knowledge acquisition in the chronic disease trajectory, rather than offering continuous value throughout all stages of the disease.

#### Theme 5: Enhanced Confidence Through Verification of Uncertain Practices

Participants in both intervention groups reported increased confidence in managing their kidney disease through the use of a chatbot. This boost in self-efficacy primarily resulted from their ability to clarify uncertain management practices and resolve ambiguities related to daily self-care decisions.

*Using the chatbot made it easier for me to practice and boosted my confidence in managing kidney disease. The ability to ask questions about things I hesitated to address allowed me to manage my condition more effectively.*

[O1]

*When I asked about something I was already doing and the chatbot confirmed it was correct, I felt relieved. However, when it advised against a certain action, I reconsidered my behavior.*

[C16]

This theme demonstrates that the value of chatbots goes beyond merely providing information; it also encompasses psychological benefits such as reduced uncertainty and enhanced self-efficacy.

## Discussion

### Principal Findings

This pilot RCT successfully met its primary goal of providing preliminary efficacy estimates and effect sizes to guide a fully powered definitive trial of personalized RAG-based chatbots for patients receiving dialysis. In this exploratory pilot, C-RAG showed a greater reduction in information needs than O-RAG in both medication and dietary domains (both Cohen *d*=−0.86, *P*=.02). Given the pilot scale, this finding should be interpreted as a preliminary signal supporting personalization as a candidate design feature, to be confirmed in a fully powered trial. When compared with standard care, C-RAG exhibited medium-to-large effect sizes (medication Cohen *d*=−0.64, dietary Cohen *d*=−0.54). Although these findings were not statistically significant in this small pilot study (n=14 per group; *P*=.13 and *P*=.55, respectively), they exceeded typical benchmarks observed in digital health interventions for chronic disease management (typically Cohen *d*=0.20‐0.35) [39], indicating potential clinical relevance pending validation in adequately powered trials. In contrast, O-RAG displayed minimal differences from the control group (medication Cohen *d*=0.13, *P*≥.99; dietary Cohen *d*=0.27, *P*=.40; diagnostic Cohen *d*=0.22, *P*≥.99), suggesting that generic RAG systems offer limited additional value over standard care.

Post hoc power analysis based on observed effect sizes suggests that a confirmatory trial with 50‐55 participants per group would achieve 80% power to detect differences between C-RAG and control at α=.05, assuming similar effect magnitudes and variances. Secondary outcomes, including kidney knowledge, self-efficacy, and adherence, did not show significant differences across groups, which aligns with the short 4-week duration suitable for pilot effect size estimation. These outcomes typically require 12‐24 weeks to demonstrate detectable changes [40] and will be primary focus areas in a future extended definitive trial.

### Mechanisms: Personalization and Information Need Fulfillment

The most striking finding was the divergent pattern of change between C-RAG and O-RAG participants. C-RAG participants showed reductions in information needs across both medication (Δ=−0.87) and dietary (Δ=−0.67) domains, indicating improved fulfillment of information needs. In contrast, O-RAG participants showed increases in the same domains (Δ=+0.96 and Δ=+0.56, respectively), while control participants showed minimal change (|Δ|<0.2). This divergence suggests that exposure to general RAG-based information may increase patients’ awareness of their unmet information needs without adequately addressing them. However, this trend did not achieve statistical significance in the current pilot sample. In contrast, personalized contextualization seems to bridge this gap by providing responses tailored to individual clinical profiles. Qualitative findings shed light on the mechanisms behind these quantitative patterns. O-RAG users who experienced perceived personalization accomplished this through iterative query refinement, which required more cognitive effort compared to the automatic contextualization offered by C-RAG. One frequent O-RAG user (who asked 89 questions) explicitly noted that his higher usage was due to the need for repeated follow-up questions to obtain answers specific to his situation. For patients with limited health literacy or lower motivation, this additional cognitive load may lead to unresolved information needs and an increased awareness of information gaps—exactly the trend observed in the O-RAG change scores.

The effect sizes (Cohen *d*=−0.86) are significantly higher than those typically seen in digital health interventions [28], underscoring the clinical importance of personalization as a design feature in AI-based patient education systems.

### Dietary Management as the Critical Unmet Need

Evidence from multiple sources consistently indicates that dietary management is the primary unmet information need among patients receiving dialysis [16,24]. In this study, dietary queries accounted for the largest single topic category in system logs, comprising 205 out of 691 (29.3%) entries, while eating-related terms appeared most frequently in keyword analysis with 133 occurrences—more than double the frequency of the next category, medication intake, which had 56 occurrences. At baseline, dietary information needs were rated highly across all 3 groups (mean scores ranged from 3.3, SD 1.1 to 3.9, SD 1.1 on a 5-point scale), reflecting a significant unmet need before any intervention. Qualitatively, 8 out of 9 interview participants identified dietary management as their most important and frequently addressed concern, regardless of group assignment.

Temporal usage patterns further illuminate when dietary information needs arise. A morning peak at 9 AM showed a significant dominance of dietary-related queries, accounting for 36% (19/53) of all inquiries, which aligns with premeal decision-making at the start of the day. Similarly, the evening peak at 9 PM also revealed that dietary queries were the most frequent category, making up 29% (14/48) of the total, indicating that patients reflect on dietary choices after dinner while managing complex nutritional restrictions. Overall, dietary information needs seem to peak around mealtimes, highlighting the importance of on-demand access to dietary guidance outside of scheduled clinical visits. This mismatch between the frequency of dietary decision-making and the periodic availability of clinical dietary guidance represents a key gap that personalized RAG systems are well-suited to address.

These findings support previous research indicating that dietary management poses the greatest self-management challenge for patients with CKD [16,41], and expand the literature by identifying the specific temporal contexts in which these information needs arise.

### Dialysis Experience Duration Drives Engagement, While Demographics Do Not

A consistent pattern observed in both quantitative and qualitative analyses was the inverse relationship between dialysis duration and chatbot utility. Patients undergoing shorter dialysis (1‐3 y) were more likely to engage with the chatbot, with 90% (9/10) classified as high users, compared with only 27.8% (5/18) of those with longer durations. Qualitative feedback indicated that patients with extensive dialysis experience (>3 y) found limited benefit from the chatbot due to redundant information, while those with shorter durations reported significant value from both systems. This trend suggests that chatbot interventions are particularly beneficial during a critical phase of knowledge acquisition in the chronic disease trajectory—specifically, the period following diagnosis when patients need to quickly learn complex self-management skills. As patients gain disease-specific expertise over time, the additional informational value of a general resource and guidance (RAG) system diminishes.

In contrast, engagement levels were not significantly associated with age (*P*=.89), proficiency with digital devices (mean 3.4, SD 1.0 vs mean 3.3, SD 0.8; *P*=.64), or prior chatbot experience (*P*=.45). This indicates that text-based chatbot systems are broadly accessible to diverse dialysis patient populations, including older adults and those without prior technology experience. This finding supports previous research suggesting that patients with CKD from various demographic backgrounds can effectively engage with digital health interventions [40]. Additionally, the observed sex pattern—where all female participants were classified as high users compared with 41.7% (10/24) of male participants (*P*=.11)—merits further investigation in larger samples, as the consistency of this pattern indicates potential sex differences in information-seeking behavior that could inform targeted implementation strategies.

### Comparison With Prior Work

The current findings contribute to the expanding literature on RAG-based chatbot systems for chronic disease management. Wang et al [27] showed that RAG augmentation improved the quality of LLM responses in diabetes education. Similarly, a recent evaluation of a custom RAG chatbot for type 2 diabetes found that evidence-based responses enhanced health literacy and patient trust. However, neither study compared personalized RAG designs with general ones, nor did they investigate whether exposure to RAG differentially impacted the fulfillment of information needs. This study addresses this gap by isolating personalization as an independent design variable, demonstrating that the method of RAG delivery—not just its presence—affects clinical outcomes. These engagement and trust patterns are consistent with human-centered perspectives on health care AI adoption, which emphasize that sustained use depends on aligning system behavior with user needs and context rather than on technical capability alone [42].

The different trajectories of information needs observed in this study have not been reported before. Previous RCTs of chatbots, such as the trial on prostate cancer by Baumgärtner et al [28] and the comparison of AI-generated vs expert-generated patient content by Lv et al [39], showed improvements in information outcomes without exploring the conditions under which chatbot exposure might inadvertently increase unmet needs. Our finding that general RAG exhibited a trend toward higher information need scores—although not statistically significant compared with the control group—suggests that the indiscriminate use of AI health information systems may not always be beneficial. While this pattern requires validation in larger samples, it presents a potentially important hypothesis with implications for implementation.

In the context of CKD literature, prior digital interventions have mainly focused on self-management behaviors and quality of life [40], with health literacy identified as a key moderator of outcomes [41]. This study complements existing research by showing that the fulfillment of information needs—a proximal outcome closely linked to health literacy—can be effectively addressed through targeted chatbot interventions, and that personalization is a crucial design element to achieve this goal. Moreover, while previous work on CKD information needs has relied on survey-based assessments [16], this study uses real-time interaction logs and qualitative interview data to provide a more detailed understanding of when and how dietary information needs arise in daily life.

### Limitations

Several limitations should be considered when interpreting these findings. First, the limited sample size and statistical power constrained definitive hypothesis testing. This pilot study enrolled 14 participants per group, which was sufficient for estimating effect sizes but underpowered for testing comparisons between C-RAG and the control. A post hoc power analysis using the observed effect sizes and variance estimates indicated a statistical power of 24% for the C-RAG vs control medication comparison (Cohen *d*=−0.64) and 19% for the dietary comparison (Cohen *d*=−0.54) at α=.05. In contrast, the better-powered C-RAG vs O-RAG comparison, with an observed power of 72% for Cohen *d*=−0.86, provides the primary basis for conclusions regarding personalization effects. The observed effect sizes offer robust estimates for planning confirmatory trials (n=50‐55 per group for 80% power) but cannot establish definitive efficacy. Additionally, the small sample size limits the reliability of subgroup analyses.

Second, the short intervention duration restricted the assessment of downstream outcomes. The 4-week period was sufficient to detect proximal changes in information needs but was inadequate for evaluating downstream effects on knowledge, self-efficacy, and adherence, which typically require 12‐24 weeks [40]. Whether reductions in information needs translate into lasting clinical benefits—such as improved laboratory values, reduced hospitalizations, and delayed progression—remains to be established through extended trials.

Third, the heterogeneity in usage introduced variance related to adherence. Individual usage ranged from 0 to 89 questions (median 13.0, IQR 4.0‐40.0), indicating significant variability in adherence that may have diminished group-level effects. This underscores the real-world challenges of implementation and highlights the need for strategies to enhance engagement in future deployments. In addition, the explicit weekly-question encouragement may have influenced usage volume and thus the engagement analyses; observed engagement should therefore be interpreted as usage under active prompting rather than spontaneous adoption.

Fourth, unmeasured moderators may have influenced the outcomes. We did not assess health literacy, which could affect both system usage and benefits [41]. Future studies should measure health literacy and explore adaptive content complexity to accommodate diverse comprehension levels. Additionally, while RAG systems reduce the risk of hallucination compared with baseline LLMs [21], occasional errors may still occur. Future implementations should incorporate uncertainty indicators, source citations, and periodic expert reviews. In addition, the community-sourced comparator responses were authentic forum answers that were not matched to the LLM outputs in length, context, or style, which may have affected the comparison’s interpretability.

Fifth, limited generalizability constrains external validity. The study was conducted at 3 tertiary hospitals in South Korea, and the enrolled sample was relatively young and digitally proficient. Findings may therefore not generalize to populations with CKD in other cultural or care settings, or to older, less technologically experienced patients, among whom uptake and benefit could differ. In addition, the Korean-language context and the CKD-specific knowledge base may hinder direct transferability to other languages, cultures, and clinical settings. Adaptation requires not only translation but also cultural customization, integration of regional guidelines, and validation by local experts. Ethical considerations—such as patient autonomy, algorithmic bias, data privacy, and accountability—also demand ongoing attention as AI systems become increasingly prevalent in clinical care. Furthermore, the phase 1 model evaluation was conducted in October 2024. Because commercial LLMs are updated frequently, the specific accuracy, safety, and readability rankings may not reflect current model versions; the contribution of phase 1 lies in the empirical, expert-based selection methodology rather than in fixed model rankings. Relatedly, the Korean-adapted readability indices (KoNLPy-based syllable and word segmentation) provided only relative comparisons across models evaluated on identical Korean text and should not be interpreted as absolute grade-level estimates, given that the underlying Flesch-based formulas were developed for English.

### Future Directions

These findings support 4 high-priority directions for future research and implementation. First, a fully powered confirmatory trial is essential. A multicenter RCT with 50‐60 participants per group and a follow-up period of 12‐24 weeks would allow for a definitive evaluation of C-RAG’s impact on important outcomes, including disease knowledge, self-efficacy, treatment adherence, and clinical parameters like serum phosphorus and potassium levels. Extended follow-up would also help determine whether reductions in information needs lead to lasting behavioral changes and improved clinical outcomes. Such trials should incorporate strategies to enhance engagement, such as personalized usage prompts and integration into clinical workflows, to address the usage variability observed in this pilot study.

Second, technical enhancements and broader population applicability are needed. Integrating food recognition technology could facilitate real-time dietary assessments, transforming C-RAG from a mere informational tool into an active behavioral support system. Automated electronic medical record connectivity would alleviate the burden of manual data entry while ensuring that responses accurately reflect the current clinical status. Additionally, adapting content complexity based on user comprehension patterns could further improve accessibility. Expanding C-RAG’s use beyond dialysis patients to include patients with earlier-stage CKD (stages 3‐4) would meet a different set of needs focused on preventing disease progression, where sustained behavioral interventions may have a greater long-term impact [17]. Furthermore, the personalized RAG framework could be applied to other complex chronic conditions that require individualized dietary and medication guidance, such as diabetes and heart failure.

Third, implementation science research is crucial for effective real-world deployment. Successful adoption depends on several factors, including clinician acceptance, workflow integration, reimbursement models, and organizational readiness. Implementation studies that use frameworks like RE-AIM (Reach, Effectiveness, Adoption, Implementation, and Maintenance) can offer actionable guidance for health care systems considering chatbot adoption, particularly regarding reach among older adults and patients with limited digital literacy.

Fourth, evaluating health economics and equity is vital for making informed adoption decisions. Cost-effectiveness analyses should investigate whether enhanced information fulfillment leads to fewer hospitalizations for electrolyte imbalances and reduced routine inquiries between visits. Additionally, assessing equity impacts is essential to ensure that AI-based interventions do not worsen existing disparities in access and outcomes, especially in underserved populations where nephrology expertise is scarce.

### Conclusions

This pilot RCT addressed its primary research question of whether personalized RAG (C-RAG) is more effective in reducing information needs than general RAG (O-RAG) and standard care, while also generating robust effect size estimates for definitive trial design. C-RAG showed a greater reduction in information needs than O-RAG, with a large effect size (Cohen *d*=−0.86) exceeding typical digital health benchmarks. Because this was a pilot trial powered for effect-size estimation rather than confirmatory testing, this result should be regarded as a preliminary estimate. When compared to standard care, C-RAG showed medium-to-large effects (Cohen *d*=−0.54 to −0.64) that, although not statistically significant in this small pilot study, provide promising preliminary estimates and critical parameters for a fully powered confirmatory trial (estimated n=50‐55 per group).

The differing trajectories of the groups highlight an important insight; general RAG systems tend to increase information needs without meeting them, though this trend needs validation through fully powered trials. In contrast, personalized systems effectively address information gaps by providing automatic clinical contextualization. Dietary management surfaced as the primary unmet need, with dialysis duration—rather than demographics or digital proficiency—being the key predictor of engagement.

These findings lay the groundwork for a definitive multicenter trial with sufficient power, extended follow-up (12‐24 weeks), and thorough outcome assessments that include knowledge, adherence, and clinical parameters. Essential next steps involve conducting a health economic evaluation, assessing equity impact in underserved populations, and testing cross-disease generalizability to determine whether personalized RAG systems can realize their potential as scalable solutions for chronic disease self-management support.

## Supplementary material

10.2196/96501 Multimedia Appendix 1 Phase 1 large language model evaluation workflow. Four large language models (ChatGPT-4o, Gemini 1.5 Flash, Claude 3.5 Sonnet, CLOVA X) were evaluated using 60 patient-generated questions sourced from KidneyCafe, an online kidney disease community. Responses were assessed by 15 clinical experts (5 nephrologists, 5 pharmacists, and 5 clinical dietitians) across three domains: inspection and diagnostics, medications and supplements, and nutrition and diet. Anonymized responses were rated on 5-point Likert scales for clinical accuracy and safety. LLM: large language model; RAG: retrieval-augmented generation.

10.2196/96501 Multimedia Appendix 2 Semistructured interview guide.

10.2196/96501 Multimedia Appendix 3 Readability scores of large language model responses by domain. Readability was assessed using three metrics: Flesch-Kincaid Grade Level (FKGL), Flesch Reading Ease (FRE), and Gunning Fog Index (GFI). ChatGPT-4o achieved the highest readability across all metrics (FKGL 9.8, FRE 62.4, GFI 11.2), corresponding to a high school reading level, compared to Claude 3.5 Sonnet, Gemini 1.5 Flash, and CLOVA X (FKGL 13.2–14.5, college level). LLM: large language model; FKGL: Flesch-Kincaid Grade Level; FRE: Flesch Reading Ease; GFI: Gunning Fog Index.

10.2196/96501 Multimedia Appendix 4 Secondary Outcomes at 4 Weeks. Secondary outcomes: group comparisons at 4 weeks for kidney disease knowledge, self-efficacy, and treatment adherence domains.

10.2196/96501 Multimedia Appendix 5 Chatbot usage patterns across temporal dimensions and content domains. (A) Topic distribution of 691 total queries, with dietary management as the predominant category (205/691, 29.3%), followed by diagnosis (22.6%) and medication (17.7%). (B) Top 15 query keywords extracted via morpheme analysis (KoNLPy Okt tokenizer), with eating-related terms and medication intake appearing most frequently. (C) Weekday usage patterns showing 1.38-fold higher engagement on weekdays compared to weekends (13.2 vs 9.6 questions per day), with peak usage from Tuesday to Thursday (14.7–17.1 questions per day). (D) Hourly usage patterns identifying 4 daily peaks at 9 AM, 2 PM, 3 PM, and 9 PM; dietary queries were most frequent at the 9 AM (36%) and 9 PM (29%) peaks. HD: hemodialysis; PD: peritoneal dialysis.

10.2196/96501 Checklist 1 CONSORT-eHEALTH checklist (V 1.6.1).

## Abbreviations

BERTScore: bidirectional encoder representations from transformers score
C-RAG: constraint retrieval-augmented generation
CKD: chronic kidney disease
FKGL: Flesch-Kincaid Grade Level
FRE: Flesch Reading Ease
GFI: Gunning Fog Index
IRB: institutional review board
KDIGO: Kidney Disease: Improving Global Outcomes
KDOQI: Kidney Disease Outcomes Quality Initiative
KiKS: Kidney Knowledge Survey
Kt/V: dialysis adequacy measure (K: urea clearance, t: time, V: volume of distribution)
LLM: large language model
MMAS-8: Morisky Medication Adherence Scale-8
MMLU: massive multitask language understanding
O-RAG: open-domain general retrieval-augmented generation
RAG: retrieval-augmented generation
RCT: randomized controlled trial
RE-AIM: Reach, Effectiveness, Adoption, Implementation, Maintenance
SUS: System Usability Scale
