# Supplementary material for: Effects of a Personalized Retrieval-Augmented Generation Chatbot on Information Needs in Patients With Chronic Kidney Disease: Mixed Methods Randomized Controlled Trial
Source: Interact J Med Res. 2026 Nov 13;15:e96501. doi: 10.2196/96501 (PMC13762686; doi:10.2196/96501)
Supplement: Supplementary file 2 [file ijmr-v15-e96501-s002.docx]

| **Category** | **Question item** |
| --- | --- |
| **1. Overall System Experience and Perceived Usefulness** | |
| **Q1** | Do you recall the process at the start of the study, when a nurse entered your health information (eg, laboratory results, medication information) into the chatbot? |
| **Q2** | Which type of chatbot do you think you used — one that provided personalized responses or one that provided general responses — and why do you think so? |
| **Q3** | If you used Type A (personalized): did the responses feel like they reflected your own health condition? If you used Type B (general): were the general responses sufficient, or were there times when you wished the response had been more personalized to your situation? |
| **2. Specific Examples of Information-Seeking Behaviors** | |
| **Q4** | What were the reasons you used the chatbot frequently, or only occasionally? |
| **Q5** | Among the diagnostic, medication, and dietary domains, which were you most curious about, and which was actually most helpful to you? Why? |
| **3. Impact of Responses on Decision-Making** | |
| **Q6** | Based on the chatbot's responses, was there anything you actually put into practice, or tried to put into practice? |
| **Q7** | After using the chatbot, did your conversations with your physician during hospital visits change in any way? |
| **Q8** | After using the chatbot, did you notice any emotional changes related to managing your condition (eg, increased confidence, increased knowledge, reduced anxiety)? |
| **4. Comparisons With Alternative Information Sources (Clinicians, Internet, and Peer Communities)** | |
| **Q9** | Before using this chatbot, through what channels (eg, healthcare providers, the internet, other patients) did you typically seek health information? |
| **5. Suggestions for Improvement** | |
| **Q10** | Was there anything about the chatbot's responses that you found unsatisfactory or lacking? |
| **Q11** | Would you be willing to continue using the chatbot in the future, and why? If your usage was low, what changes might encourage you to use it more? |
| **Q12** | Did you experience any inconvenience related to using the chatbot through a smartphone? |
| Q13 | Is there anything else about your experience using the chatbot that you would like to share? |

**Multimedia Appendix 5. Semi-Structured Interview Guide.** C-RAG: constraint retrieval-augmented generation; O-RAG: open-domain retrieval-augmented generation.
