# Supplementary material for: Effects of a Personalized Retrieval-Augmented Generation Chatbot on Information Needs in Patients With Chronic Kidney Disease: Mixed Methods Randomized Controlled Trial
Source: Interact J Med Res. 2026 Nov 13;15:e96501. doi: 10.2196/96501 (PMC13762686; doi:10.2196/96501)
Supplement: Supplementary file 3 [file ijmr-v15-e96501-s004.docx]

| **Outcome** | **C-RAG  Mean (SD)** | **O-RAG  Mean (SD)** | **Control  Mean (SD)** | **ANCOVA  F (p value)** |
| --- | --- | --- | --- | --- |
| KiKS | 18.93 (3.50) | 19.71 (3.27) | 19.64 (2.24) | F=0.182, p=0.834 |
| CKD  Self efficacy | 83.43 (14.51) | 90.86 (16.37) | 86.00 (11.37) | F=0.923, p=0.406 |
| Medication Adherence | 5.62 (1.32) | 5.39 (1.34) | 5.36 (1.46) | F=0.175, p=0.840 |
| Dietary Adherence | 2.79 (0.58) | 3.43 (0.76) | 3.14 (0.66) | F=0.906, p=0.413 |
| Fluid Adherence | 2.93 (0.73) | 3.29 (0.83) | 2.93 (0.73) | F=0.002, p=0.998 |

**Multimedia Appendix 3**. **Secondary Outcomes at 4 Weeks.** Secondary outcomes: group comparisons at 4 weeks for kidney disease knowledge, self-efficacy, and treatment adherence domains.
